# The Silent Cognitive Burden of Chronic Pain: Protocol for an AI-Enhanced Living Dose–Response Bayesian Meta-Analysis

**DOI:** 10.3390/jcm14197030

**Published:** 2025-10-04

**Authors:** Kevin Pacheco-Barrios, Rafaela Machado Filardi, Edward Yoon, Luis Fernando Gonzalez-Gonzalez, Joao Victor Ribeiro, Joao Pedro Perin, Paulo S. de Melo, Marianna Leite, Luisa Silva, Alba Navarro-Flores

**Affiliations:** 1 Neuromodulation Center and Center for Clinical Research Learning, Spaulding Rehabilitation Hospital and Massachusetts General Hospital, Harvard Medical School, Boston, MA 02141, USAjvgomes@mgb.org (J.V.R.); aluisasilva90@gmail.com (L.S.); 2 Unidad de Investigación para la Generación y Síntesis de Evidencias en Salud, Vicerrectorado de Investigación, Universidad San Ignacio de Loyola, Lima 150114, Peru; 3 Admission AG, Irvine, CA 92618, USA; 4 Department of Neurology, Santa Marcelina School of Medicine, São Paulo 05006-020, Brazil; 5 Institute of Psychiatric Phenomics and Genomics (IPPG), LMU University Hospital, LMU Munich, 80336 Munich, Germany; 6 International Max Planck Research School for Translational Psychiatry (IMPRS-TP), 82152 Munich, Germany

**Keywords:** chronic pain, cognitive decline, Bayesian meta-analysis, living systematic review, AI-aided evidence synthesis

## Abstract

**Background:** Chronic pain affects nearly one in five adults worldwide and is increasingly recognized not only as a disease but as a potential risk factor for neurocognitive decline and dementia. While some evidence supports this association, existing systematic reviews are static and rapidly outdated, and none have leveraged advanced methods for continuous updating and robust uncertainty modeling. **Objective:** This protocol describes a living systematic review with dose–response Bayesian meta-analysis, enhanced by artificial intelligence (AI) tools, to synthesize and maintain up-to-date evidence on the prospective association between any type of chronic pain and subsequent cognitive decline. **Methods:** We will systematically search PubMed, Embase, Web of Science, and preprint servers for prospective cohort studies evaluating chronic pain as an exposure and cognitive decline as an outcome. Screening will be semi-automated using natural language processing models (ASReview), with human oversight for quality control. Bayesian hierarchical meta-analysis will estimate pooled effect sizes and accommodate between-study heterogeneity. Meta-regression will explore study-level moderators such as pain type, severity, and cognitive domain assessed. If data permit, a dose–response meta-analysis will be conducted. Living updates will occur biannually using AI-enhanced workflows, with results transparently disseminated through preprints and peer-reviewed updates. **Results:** This is a protocol; results will be disseminated in future reports. **Conclusions:** This living Bayesian systematic review aims to provide continuously updated, methodologically rigorous evidence on the link between chronic pain and cognitive decline. The approach integrates innovative AI tools and advanced meta-analytic methods, offering a template for future living evidence syntheses in neurology and pain research.

## 1. Introduction

Chronic pain is one of the most prevalent (around 30%) and costly health conditions worldwide, with profound consequences for physical functioning, psychological well-being, and social participation [1]. Defined as pain persisting for more than three months, chronic pain is a leading cause of disability and, despite advances in pain management, many individuals continue to experience persistent pain that substantially impairs quality of life [2].

Recent research has shifted attention to the cognitive and neurological consequences of chronic pain, revealing that persistent pain may extend its impact beyond somatic discomfort to influence brain structure and function [3]. Neuroimaging studies have shown that chronic pain is associated with alterations in brain networks responsible for memory, attention, and executive function, including the prefrontal cortex connectivity, hippocampal circuits, and the default mode network [4,5]. These findings suggest that the pain experience may accelerate neurocognitive decline, potentially increasing the risk of mild cognitive impairment (MCI) and dementia, possibly via alterations in brain connectivity and structure, neuroinflammatory processes, autonomic dysregulation, psychosocial determinants, and changes in the microbiome [6].

Understanding this link is of high public health importance, given the aging global population and the rising incidence of neurodegenerative disorders. Identifying chronic pain as a modifiable risk factor for cognitive decline could have significant implications for prevention strategies and clinical practice. Despite this relevance, evidence on the temporal association between chronic pain and cognitive decline remains inconsistent and fragmented. While some longitudinal studies suggest that individuals with chronic pain have a higher risk of subsequent cognitive impairment [7], the directionality of this association is still unclear [8].

Existing systematic reviews have attempted to synthesize this body of literature, but they face limitations mostly due to the need of establishing causality or directionality, requiring a larger number of longitudinal studies [9]. Additionally, the potential long-term dose effects of pain severity in cognition remained unexplored. Recent evidence from a meta-analysis of chronic back pain in older adults shows its relationship with memory-related diseases and subjective cognitive decline in populations from the USA and Europe [10]. The mediator that increased cognitive decline was alcohol intake, and the ones relieving it were smoking cessation, physical activity, and proper sleeping habits. Another review focusing on women with endometriosis and chronic pelvic pain found only seven studies reporting cognitive scores, and only two of them reported an association with cognitive decline, showing a need for studies in this field [11]. New primary evidence shows the effects of moderate-to-severe chronic pain in reducing executive functioning in middle-aged adult men during the Vietnam era, which highlights the importance of including pain severity in the analysis of these effects [12].

Furthermore, static reviews become outdated as new cohort data emerge, hindering timely updates to clinical guidelines and policy decisions. In parallel, methodological advances in evidence synthesis offer promising solutions to these challenges. Living systematic reviews (LSRs) maintain continuous updates to incorporate new evidence as it becomes available, ensuring that conclusions remain current and relevant [13]; moreover, LSRs are particularly critical for dynamically updating guidelines and public health recommendations. Bayesian meta-analyses provide a flexible framework for combining evidence, allowing incorporation of prior knowledge and direct probabilistic interpretation of results [14]. Moreover, prospective studies with Bayesian modeling, including meta-analyses, although not a substitute for randomized controlled studies, can enhance the confidence of causal inference from observational studies. Additionally, artificial intelligence (AI) and natural language processing (NLP) models have shown potential to enhance efficiency in systematic reviews, particularly for citation screening and data extraction, by reducing manual workload and improving consistency [15].

Given these opportunities, we propose a living systematic review with dose–response Bayesian meta-analysis to evaluate the association between chronic pain and subsequent cognitive decline, with an accumulative approach for updating the evidence. This project integrates a novel methodology with a clinically important question, aiming to produce a continuously updated, robust, and transparent synthesis of the best available evidence. By leveraging AI tools (ASReview, version 2.0) for semi-automated screening and data extraction, we aim to streamline the review process while maintaining high methodological rigor through human oversight. The Bayesian approach will enable dynamic updating of effect estimates, providing nuanced insights into the relationship between chronic pain and cognitive health as new studies are published.

Ultimately, this living evidence synthesis will inform healthcare professionals, researchers, and policymakers about the potential cognitive risks associated with chronic pain and highlight opportunities for early intervention and preventive care. The protocol presented here outlines our planned methods, including eligibility criteria, search strategies, screening processes, statistical modeling, living update procedures, and plans for dissemination. We believe that this research will not only clarify an important aspect of pain research but also serve as a model for future living reviews that incorporate AI and advanced statistical methods to address pressing public health questions.

## 2. Methods

The report of this protocol will follow the PRISMA-P guidelines [16]. For this systematic review with meta-analysis we will follow the Preferred Reporting Items for Systematic Reviews and Meta-Analysis (PRISMA) guidelines [17]. The protocol was registered in PROSPERO.

### 2.1. Eligibility Criteria

#### 2.1.1. Primary Studies

We will include only longitudinal cohorts that provide enough information for the calculation of the proportions of interest. No restrictions regarding country of origin or language will apply.

#### 2.1.2. Participants

Participants will be adults with a diagnosed chronic pain condition of any etiology and severity, including primary and secondary chronic pain conditions [18]. The setting of the studies could be communitarian or institutional, and special groups like military and student populations could be included. We will use a pragmatic definition of chronic pain, which is pain that persists or recurs for longer than three months [18].

#### 2.1.3. Primary and Secondary Outcomes

The primary outcome will be the subsequent development of cognitive decline (mild cognitive impairment or dementia) defined by standardized scales. Secondary outcomes will include the cognitive scores from validated scales and assessments of cognitive domains (attention, executive functions, memory, language, visuospatial, social cognition, and self-awareness).

### 2.2. Systematic Search Strategy

The search will be conducted in October 2025. We will search four databases since their inception date: PubMed, Web of Science, and Embase. Additionally, we will search the list of references of the recent related systematic reviews [9,19,20,21].

The search strategy was designed originally for PubMed using the following key terms and their synonyms: chronic pain, dementia, and longitudinal studies. Then, it was adapted to the syntax of the other databases (Table 1). For the verification of the search efficacy, we included relevant articles as sentinels.

### 2.3. Data Records and Screening Process

All retrieved citations will be imported into EndNote X9 for initial de-duplication using Bramer’s method [22], followed by export into the ASReview LAB platform for semi-automated screening. ASReview is an open-source active learning framework that uses machine learning algorithms to prioritize records most likely to be relevant [23]. We will employ the default feature extraction method, term frequency–inverse document frequency (TF–IDF) vectorization, in combination with a linear support vector machine (SVM) classifier. This model has been shown to perform well in prioritizing biomedical literature, achieving high recall with fewer records screened [23]. To ensure robustness in a domain with multilingual literature and diverse pain-related terminology, we will address potential challenges by (a) enriching the search strings with validated synonyms from MeSH and UMLS ontologies, (b) translating non-English abstracts using machine translation tools for inclusion in the AI prioritization workflow, and (c) harmonizing terminology across datasets to minimize misclassification.

At the outset, two reviewers will manually screen a random training set of at least 100 records, labeled as “relevant” or “irrelevant.” These labels will be used to iteratively train the model, which will then rank the remaining records by predicted relevance. The screening process will proceed in cycles: after each batch of 50–100 records is screened, the model will retrain, updating its predictions and refining the priority list. This iterative approach ensures that potentially eligible studies are identified early, reducing screening workload.

To ensure methodological rigor, we will set a stopping criterion when 95% estimated recall is reached, based on the model’s learning curve and plateau in new inclusions over several iterations. All records ranked below the stopping threshold will be randomly sampled (at least 5% of the remaining pool) and double-screened to confirm that no eligible studies are missed. The reasons for exclusion will be annotated.

#### Benchmarking and Quality Assurance

To evaluate and calibrate the AI-assisted screening process, we will construct a gold-standard set (GSS) comprising the union of studies included in previous systematic reviews of chronic pain and cognitive decline (search up to January 2022) [9,19,20,21]. The GSS will be deduplicated and restricted to prospective cohort designs that match our PICO criteria. Using ASReview in simulation (oracle) mode, we will benchmark multiple prioritization models (TF–IDF + linear SVM, logistic regression, multinomial naïve Bayes, and a sentence-embedding model) with identical seed papers. Performance will be assessed using Recall@N, Work Saved over Sampling at 95% recall (WSS@95), precision–recall AUC, and time-to-discovery curves. The highest-performing configuration will be pre-specified for the live screen. During prospective screening, we will apply a conservative stopping rule (learning-curve plateau and estimated recall ≥ 95%) and dual-screen a 5–10% random sample of low-ranked exclusions to estimate false-negative rates. All benchmarking code, metrics, and learning-curve plots will be archived and reported.

During the active learning training phase, we will calculate inter-rater agreement metrics (Cohen’s κ) between AI predictions and human reviewer decisions on the calibration set. These values will be reported in the protocol update and subsequent publications to quantify alignment between the algorithm and human reviewers. In addition, precision, recall, and learning-curve plots will be reported for transparency. This layered validation strategy will ensure that domain-specific terminology and language diversity do not compromise sensitivity and that AI performance is transparently benchmarked against human expert standards.

### 2.4. Data Extraction

Two independent authors will conduct this step using a previously designed database. Relevant variables related to different aspects will be extracted:

Methodological: Study design (prospective, retrospective), type of sampling (census, randomized, convenience), detection method (medical records, self-report).

Epidemiological: Country of the study, income category, type of population (general population, military, etc.), age and sex of the participants, recruitment setting (community, institutional), educational level, marital status, employment status, ethnicity.

Clinical variables: Chronic pain definition (including time definition and etiology), presence of physical comorbidities (cardiovascular, metabolic, other degenerative disorders), presence of neuropsychiatric comorbidities (depression, anxiety, Parkinson’s disease, etc.), severity of pain, received treatments. In order to harmonize the different definitions of cognitive decline according to the various cognitive tests available, we will categorize this variable as dichotomous (present vs. absent) using the cut-offs established in each cognitive test. Cognitive decline as a continuous variable will be constructed using the percentiles of the scores based on the developers’ manuals.

Outcomes: Sample size, number of patients with chronic pain, number of patients with dementia, cognitive scores before and after follow-up.

In the case of incomplete or dubious information, the corresponding authors will be contacted up to three times via email. In case of no response and insufficient data for extraction, the studies will be excluded.

#### Effect Measures

The main effect measure of interest is the association between chronic pain and cognitive decline (mild cognitive impairment or dementia), which will be reported as risk ratios (RRs) or hazard ratios (HRs) with their 95% credibility intervals, according to the design of the included studies. For the secondary outcomes, mean difference and 95% credibility intervals will be reported from changes in cognitive scores.

### 2.5. Risk of Bias and Evidence Certainty Assessment

The risk of bias will be assessed using the Risk Of Bias In Non-randomized Studies—of Exposure (ROBINS-E, version 1.0) tool [24]. We will evaluate the certainty of the evidence using the Grading of Recommendations, Assessment, Development, and Evaluation (GRADE) Working Group criteria [25].

### 2.6. Data Synthesis

A narrative synthesis will be conducted first, describing the basic characteristics of the populations, the studies’ design, and the variables of interest.

We will conduct a Bayesian hierarchical random-effects meta-analysis to estimate pooled effect sizes (hazard ratios or relative risks) for the association between chronic pain and cognitive decline. This model accounts for between-study heterogeneity by assuming that true effect sizes are drawn from a distribution, with study-specific estimates modeled as random deviations [26]. Priors for the overall mean effect will be weakly informative normal distributions centered at zero (mean log effect = 0, SD = 1). This decision provides mild regularization, reducing the risk of extreme or implausible estimates in sparse data settings while not constraining the analysis to strong prior beliefs [27]. And priors for the heterogeneity parameter (τ) will follow a half-Cauchy distribution with a scale of 0.5. The half-Cauchy (0.5) prior on heterogeneity is recommended in Bayesian meta-analysis as it reflects empirical distributions of τ^2^ observed across hundreds of meta-analyses, offering a realistic but not overly diffuse expectation of heterogeneity [14,27]. We will fit models using Hamiltonian Monte Carlo sampling implemented in *Stan* (version 2.32.7) via the *brms* (version 2.22.0) R package [28], running 4 chains with 4000 iterations each, discarding the first 1000 as warm-up. Convergence will be assessed using the R-hat statistic (<1.01) and visual inspection of trace plots. Results will be presented as posterior means with 95% credible intervals, and posterior probabilities of clinically meaningful effects (e.g., RR > 1.2) will be calculated.

A secondary analysis will include the identification of associated factors to subsequent cognitive decline: (a) clinical: presence of physical comorbidities, mental health comorbidities, chronic pain duration, chronic pain type, received treatments; and (b) epidemiological: age, sex, socioeconomic status, educational level, employment status. If sufficient studies are included in the main outcome meta-analysis (minimum 10 studies), a meta-regression analysis will be conducted using the meta-reg function. The R^2^ (proportion of heterogeneity explained), tau^2^ (residual heterogeneity), and QM (moderator significance test) will be reported.

The assessment of the statistical heterogeneity will be conducted using the chi-square test and the I^2^ statistic, and a value lower than 40% will correspond with not substantial heterogeneity [25]. The publication bias will be assessed, if more than 10 studies are included, by visually inspecting a funnel plot and statistical evaluation with the Egger test [29].

If sufficient data are available (≥3 studies reporting ≥3 exposure levels or continuous measures of pain severity/duration), we will conduct a Bayesian dose–response meta-analysis (DRMA) to quantify the functional relationship between chronic pain intensity and cognitive decline risk. Continuous pain exposure will be modeled using restricted cubic splines with 3–5 knots placed at percentiles of the exposure distribution [30,31]. This approach allows for flexible, non-linear associations while preserving interpretability. Priors for spline coefficients will be set as weakly informative normal distributions (mean = 0, SD = 2). Both linear and non-linear models will be compared using leave-one-out cross-validation [32] to determine best fit. If the data are insufficient for non-linear modeling but at least two distinct exposure categories are available, we will fit a simplified linear trend model to estimate the average incremental risk per unit or category increase in pain severity/duration. If harmonization of exposure categories across studies is not possible, we will perform a narrative synthesis, supported by descriptive tables summarizing the exposure definitions, categories, and reported cognitive outcomes. This fallback hierarchy will ensure that all available evidence on severity or duration gradients is captured and transparently reported. Analyses will be implemented using the *rstanarm* (version 2.32.1) and *dosresmeta* (version 2.2.0) packages in R.

All analyses will be conducted in R (version 4.3.0 or later). Reproducibility will be ensured through the use of R Markdown workflows, with all code and analytic outputs deposited in an open-access repository. Model convergence, prior sensitivity analyses, and predictive checks will be systematically reported.

### 2.7. Living Update Process

**Figure 1:**
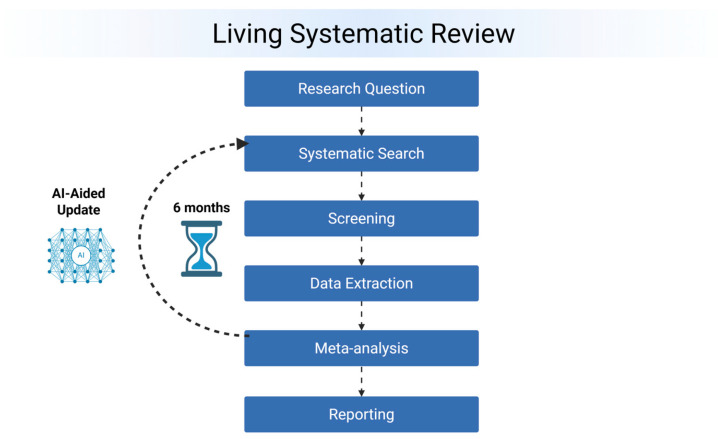
Living systematic review workflow.

The living systematic review will be updated biannually. At each update, automated database queries will retrieve new citations, which will be screened and extracted using an AI-enhanced workflow with ASReview. Newly identified studies will be extracted following the reported approach above. The new effect estimates will be incorporated into the Bayesian model, with the posterior from the previous iteration serving as the prior for the update [33]. This cumulative evidence synthesis ensures that conclusions evolve in light of new data. Version control will be maintained using GitHub (version 3.15.12), with all data, code, and model outputs timestamped and archived. Peer-reviewed updates will be published in indexed journals as brief communications. Additionally, a public-facing dashboard will display current pooled estimates, trends over time, and update histories. This process is visually explained in Figure 1.

## 3. Discussion

Chronic pain is increasingly recognized as more than a symptom of underlying pathology; it is a complex, multifactorial condition with potential systemic consequences, including neurocognitive decline. The present protocol addresses an important evidence gap by synthesizing prospective cohort data through a continuously updated, AI-augmented, Bayesian meta-analytic framework.

### 3.1. Potential Causal Pathways

**Figure 2:**
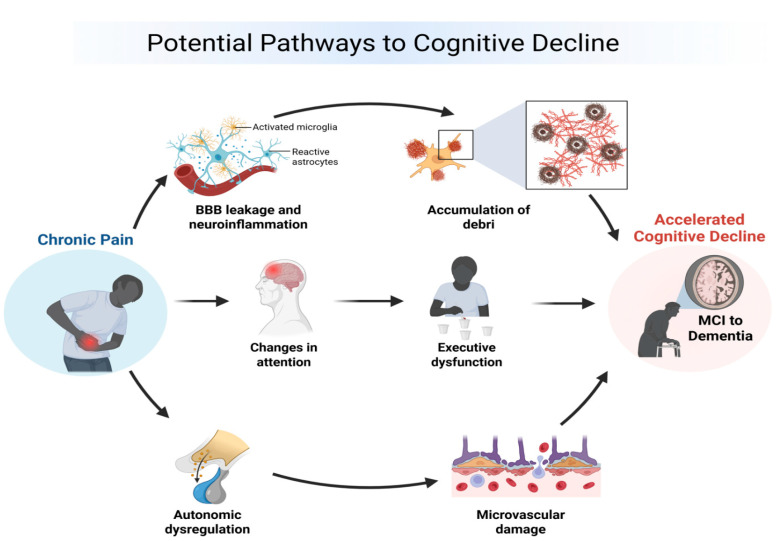
Potential causal links between chronic pain and cognitive decline.

Three plausible mechanistic pathways may underline the observed association between chronic pain and cognitive decline (Figure 2). First, persistent nociceptive input may trigger chronic neuroinflammation, leading to activation of microglia and astrocytes [34]. Sustained neuroinflammation can impair the glymphatic system’s clearance of metabolic waste, including amyloid-β and tau proteins, resulting in the accumulation of neurotoxic debris that accelerates neurodegenerative processes [35,36]. Second, chronic pain has been shown to disrupt attentional networks and reduce cognitive resources allocated to executive control, possibly through altered activity and connectivity within the prefrontal cortex and anterior cingulate cortex [37,38]. Over time, this attentional and executive dysfunction may precipitate measurable cognitive decline. Third, chronic pain is often associated with autonomic dysregulation, including heightened sympathetic tone and reduced parasympathetic activity [39]. Such imbalance can impair cerebral autoregulation, promote microvascular damage, and contribute to microinfarctions or altered brain perfusion, all of which are linked to cognitive deterioration [40].

### 3.2. New Approaches in Evidence Synthesis

Emerging fields like pain-related cognitive neuroscience evolve rapidly, with new cohort studies regularly published. Although there are two previous systematic reviews examining chronic pain (as a broader term) and cognitive impairment/dementia, they reported limitations in their development of them. The inclusion of different pain subtypes was challenging due to the lack of detailed description used in the primary studies, the different ratings of chronic pain used hampered the dose–response evaluation of the association, and the lack of certainty assessment using GRADE reduces the interpretability of their findings [9]. For the other review, the heterogeneity of reporting pain severity and chronic pain subtype and the lack of screening for dementia at baseline or further evaluation of confounders in pooled estimates were the most burdensome limitations [20]. The limitations reported showed that the current evidence synthesis is still insufficient to causally infer a relationship between chronic pain and dementia, and that a new synthesis is needed for further exploration of this association. Static systematic reviews risk becoming outdated within months, leading to gaps in evidence translation. A living systematic review (LSR) framework ensures that clinical and policy decisions are informed by the most current data, supporting timely integration into practice guidelines [13]. Bayesian methods offer distinct advantages in the LSR context, enabling the seamless incorporation of new evidence into prior posterior distributions without the need for de novo analyses at each update [33]. This continuous learning process provides more nuanced quantification of uncertainty and facilitates probabilistic interpretations directly relevant to clinical decision making.

### 3.3. Strengths and Limitations

Our design has four notable strengths: (1) inclusion of only prospective cohort studies to strengthen temporal inference; (2) AI-enhanced workflows, improving efficiency in literature screening and data extraction while maintaining rigorous human oversight; (3) robust Bayesian hierarchical modeling to accommodate heterogeneity and enable flexible subgroup and meta-regression analyses; and (4) planned dose–response modeling to investigate potential exposure–response gradients.

We recognize several potential challenges. Despite restricting our synthesis to prospective cohort studies, the risk of residual confounding and bidirectional causality cannot be fully excluded. Chronic pain and cognitive decline share overlapping biological and psychosocial determinants, including depression, sleep disruption, and cardiovascular comorbidities, which may independently influence both conditions [7,41]. Moreover, early cognitive impairment may alter pain perception, coping mechanisms, or reporting accuracy, leading to potential reverse causality. While adjusted estimates and stratified analyses in primary studies mitigate some bias, heterogeneity in adjustment strategies limits full control of confounding. Consequently, the findings should be interpreted as evidence of association rather than definitive proof of causation, underscoring the need for future mechanistic and interventional studies to clarify causal pathways. Moreover, variability in definitions and measures of chronic pain and cognitive decline may introduce heterogeneity, necessitating careful harmonization and sensitivity analyses. AI-assisted screening and extraction, while efficient, require stringent quality assurance and benchmarking to ensure that sensitivity and precision remain within acceptable thresholds [15]. Additionally, sustaining the living component demands ongoing resources and coordination.

### 3.4. Potential Impact

The anticipated outputs of this review extend beyond quantifying the association between chronic pain and cognitive decline. They include identifying key effect modifiers, informing mechanistic hypotheses, and guiding clinical screening for cognitive impairment in pain populations. By maintaining an up-to-date synthesis, our findings will be positioned to influence global health practice guidelines, shape preventive strategies, and stimulate targeted mechanistic research.

## 4. Conclusions

This protocol outlines an innovative, AI-enhanced, Bayesian living systematic review to assess the prospective association between chronic pain and cognitive decline. By integrating advanced analytic methods, continuous evidence updating, and mechanistic framing, the project aims to provide clinicians, researchers, and policymakers with the most rigorous and current synthesis available, increase our understanding regarding the potential consequences of chronic pain in cognition, and guide the elaboration and update of clinical practice guidelines, ultimately contributing to improved patient outcomes and public health strategies to prevent dementia.

## Figures and Tables

**Table 1:** Systematic search.

Database	Search
Pubmed	((((Pain/OR Pain Perception/OR Chronic Pain/OR Neuralgia/OR ((Chronic) adj6 Pain* OR headache* OR Cephalalgia* OR Cephalodynia* OR neuropath* OR Arthritis/OR Back Pain/OR Complex Regional Pain Syndromes/OR Facial Neuralgia/OR Femoral Neuropathy/OR Fibromyalgia/OR Headache/OR Headache Disorders/OR Low Back Pain/OR Migraine Disorders/OR Musculoskeletal Pain/OR Myalgia/OR Myofascial Pain Syndromes/OR Neck Pain/OR Neuralgia/OR Osteoarthritis/OR Phantom Limb/OR Post-Traumatic Headache/OR Radial Neuropathy/OR Tension-type Headache/OR Trigeminal Autonomic Cephalalgias/) OR ((pain[TIAB]) AND (arthrit*[TIAB] OR “complex regional pain”[TIAB] OR CRPS[TIAB] OR fibromyalgia[TIAB] OR headache*[TIAB] OR “low back pain”[TIAB] OR migrain*[TIAB] OR myalgia[TIAB] OR neuralgia[TIAB] or neuropath*[TIAB] OR nocicept*[TIAB] OR osteoarthrit*[TIAB] OR osteo-arthrit*[TIAB] OR “phantom limb”[TIAB] OR “reflex sympathetic dystrophy”[TIAB])) OR (central pain[TIAB] OR central sensitization[TIAB]))) AND ((Cognitive Dysfunction/OR Dementia/OR “Cognitive decline”[TIAB] OR “cognitive impairment”[TIAB] OR dementia [TIAB]))) AND ((((Epidemiologic Studies/OR Case–Control Studies/OR Cohort Studies/OR Cross-Sectional Studies/OR “case control” [TIAB] OR cohort [TIAB] OR prevalence[TIAB] OR incidence[TIAB]) AND (study [TIAB] OR studies [TIAB] OR analys*[TIAB] OR “follow up” [TIAB] OR observational [TIAB] OR uncontrolled [TIAB] OR non-randomized [TIAB] OR nonrandomized [TIAB] OR non randomized [TIAB] OR nonrandomized [TIAB] OR epidemiologic* [TIAB]))))) NOT (((letter [pt] OR editorial [pt] OR news [pt] OR historical article [pt] OR case reports [pt] OR letter [TI] OR comment* [TI] OR animal* [TI] OR “Animal Experimentation” [Mesh] OR “Animal Experimentation” [Mesh] OR “Rodentia” [Mesh] OR rats [TI] OR rat [TI] OR mouse [TI] OR mice [TI])))
Embase	((((Pain/de OR ‘Pain Perception’/de OR ‘Chronic Pain’/de OR Neuralgia/de OR ((Chronic) NEAR/6 Pain* OR headache* OR Cephalalgia* OR Cephalodynia* OR neuropath* OR Arthritis/de OR ‘Back Pain’/de OR ‘Complex Regional Pain Syndromes’/de OR ‘Facial Neuralgia’/de OR ‘Femoral Neuropathy’/de OR Fibromyalgia/de OR Headache/de OR ‘Headache Disorders’/de OR ‘Low Back Pain’/de OR ‘Migraine Disorders’/de OR ‘Musculoskeletal Pain’/de OR Myalgia/de OR ‘Myofascial Pain Syndromes’/de OR ‘Neck Pain’/de OR Neuralgia/de OR Osteoarthritis/de OR ‘Phantom Limb’/de OR ‘Post-Traumatic Headache’/de OR ‘Radial Neuropathy’/de OR ‘Tension-type Headache’/de OR ‘Trigeminal Autonomic Cephalalgias’/de) OR ((pain:ti,ab) AND (arthrit*:ti,ab OR ‘complex regional pain’:ti,ab OR CRPS:ti,ab OR fibromyalgia:ti,ab OR headache*:ti,ab OR ‘low back pain’:ti,ab OR migrain*:ti,ab OR myalgia:ti,ab OR neuralgia:ti,ab OR neuropath*:ti,ab OR nocicept*:ti,ab OR osteoarthrit*:ti,ab OR osteo-arthrit*:ti,ab OR ‘phantom limb’:ti,ab OR ‘reflex sympathetic dystrophy’:ti,ab)) OR (‘central pain’:ti,ab OR ‘central sensitization’:ti,ab))) AND ((‘Cognitive Dysfunction’/de OR Dementia/de OR ‘Cognitive decline’:ti,ab OR ‘cognitive impairment’:ti,ab OR dementia:ti,ab))) AND ((((‘Epidemiologic Studies’/de OR ‘Case–Control Studies’/de OR ‘Cohort Studies’/de OR ‘Cross-Sectional Studies’/de OR ‘case control’:ti,ab OR cohort:ti,ab OR prevalence:ti,ab OR incidence:ti,ab) AND (study:ti,ab OR studies:ti,ab OR analys*:ti,ab OR ‘follow up’:ti,ab OR observational:ti,ab OR uncontrolled:ti,ab OR non-randomized:ti,ab OR nonrandomized:ti,ab OR ‘non randomized’:ti,ab OR nonrandomized:ti,ab OR epidemiologic*:ti,ab))))) NOT (((term:it OR term:it OR term:it OR term:it OR term:it OR letter:ti OR comment*:ti OR animal*:ti OR ‘Animal Experimentation’/exp OR ‘Animal Experimentation’/exp OR Rodentia/exp OR rats:ti OR rat:ti OR mouse:ti OR mice:ti)))
Central	(((([mh ^Pain] OR [mh ^“Pain Perception”] OR [mh ^“Chronic Pain”] OR [mh ^Neuralgia] OR ((Chronic) NEAR/6 Pain* OR headache* OR Cephalalgia* OR Cephalodynia* OR neuropath* OR [mh ^Arthritis] OR [mh ^“Back Pain”] OR [mh ^“Complex Regional Pain Syndromes”] OR [mh ^“Facial Neuralgia”] OR [mh ^“Femoral Neuropathy”] OR [mh ^Fibromyalgia] OR [mh ^Headache] OR [mh ^“Headache Disorders”] OR [mh ^“Low Back Pain”] OR [mh ^“Migraine Disorders”] OR [mh ^“Musculoskeletal Pain”] OR [mh ^Myalgia] OR [mh ^“Myofascial Pain Syndromes”] OR [mh ^“Neck Pain”] OR [mh ^Neuralgia] OR [mh ^Osteoarthritis] OR [mh ^“Phantom Limb”] OR [mh ^“Post-Traumatic Headache”] OR [mh ^“Radial Neuropathy”] OR [mh ^“Tension-type Headache”] OR [mh ^“Trigeminal Autonomic Cephalalgias”]) OR ((pain:ti,ab) AND (arthrit*:ti,ab OR “complex regional pain”:ti,ab OR CRPS:ti,ab OR fibromyalgia:ti,ab OR headache*:ti,ab OR “low back pain”:ti,ab OR migrain*:ti,ab OR myalgia:ti,ab OR neuralgia:ti,ab OR neuropath*:ti,ab OR nocicept*:ti,ab OR osteoarthrit*:ti,ab OR osteo-arthrit*:ti,ab OR “phantom limb”:ti,ab OR “reflex sympathetic dystrophy”:ti,ab)) OR (“central pain”:ti,ab OR “central sensitization”:ti,ab))) AND (([mh ^“Cognitive Dysfunction”] OR [mh ^Dementia] OR “Cognitive decline”:ti,ab OR “cognitive impairment”:ti,ab OR dementia:ti,ab))) AND (((([mh ^“Epidemiologic Studies”] OR [mh ^“Case–Control Studies”] OR [mh ^“Cohort Studies”] OR [mh ^“Cross-Sectional Studies”] OR “case control”:ti,ab OR cohort:ti,ab OR prevalence:ti,ab OR incidence:ti,ab) AND (study:ti,ab OR studies:ti,ab OR analys*:ti,ab OR “follow up”:ti,ab OR observational:ti,ab OR uncontrolled:ti,ab OR non-randomized:ti,ab OR nonrandomized:ti,ab OR “non randomized”:ti,ab OR nonrandomized:ti,ab OR epidemiologic*:ti,ab))))) NOT (((letter:pt OR editorial:pt OR news:pt OR “historical article”:pt OR “case reports”:pt OR letter:ti OR comment*:ti OR animal*:ti OR [mh “Animal Experimentation”] OR [mh “Animal Experimentation”] OR [mh Rodentia] OR rats:ti OR rat:ti OR mouse:ti OR mice:ti)))
Web of Science	((((Pain OR “Pain Perception” OR “Chronic Pain” OR Neuralgia OR ((Chronic) NEAR/6 Pain* OR headache* OR Cephalalgia* OR Cephalodynia* OR neuropath* OR Arthritis OR “Back Pain” OR “Complex Regional Pain Syndromes” OR “Facial Neuralgia” OR “Femoral Neuropathy” OR Fibromyalgia OR Headache OR “Headache Disorders” OR “Low Back Pain” OR “Migraine Disorders” OR “Musculoskeletal Pain” OR Myalgia OR “Myofascial Pain Syndromes” OR “Neck Pain” OR Neuralgia OR Osteoarthritis OR “Phantom Limb” OR “Post-Traumatic Headache” OR “Radial Neuropathy” OR “Tension-type Headache” OR “Trigeminal Autonomic Cephalalgias”) OR ((pain) AND (arthrit* OR “complex regional pain” OR CRPS OR fibromyalgia OR headache* OR “low back pain” OR migrain* OR myalgia OR neuralgia OR neuropath* OR nocicept* OR osteoarthrit* OR osteo-arthrit* OR “phantom limb” OR “reflex sympathetic dystrophy”)) OR (“central pain” OR “central sensitization”))) AND ((“Cognitive Dysfunction” OR Dementia OR “Cognitive decline” OR “cognitive impairment” OR dementia))) AND ((((“Epidemiologic Studies” OR “Case–Control Studies” OR “Cohort Studies” OR “Cross-Sectional Studies” OR “case control” OR cohort OR prevalence OR incidence) AND (study OR studies OR analys* OR “follow up” OR observational OR uncontrolled OR non-randomized OR nonrandomized OR “non randomized” OR nonrandomized OR epidemiologic*))))) NOT (((letter OR editorial OR news OR “historical article” OR “case reports” OR letter OR comment* OR animal* OR “Animal Experimentation” OR “Animal Experimentation” OR Rodentia OR rats OR rat OR mouse OR mice)))

## Data Availability

Data is contained within the article.

## References

[1] Cohen S.P., Vase L., Hooten W.M. (2021). Chronic pain: An update on burden, best practices, and new advances. Lancet.

[2] Rice A.S.C., Smith B.H., Blyth F.M. (2016). Pain and the global burden of disease. Pain.

[3] Malfliet A., Coppieters I., Van Wilgen P., Kregel J., De Pauw R., Dolphens M., Ickmans K. (2017). Brain changes associated with cognitive and emotional factors in chronic pain: A systematic review. Eur. J. Pain.

[4] Apkarian V.A., Hashmi J.A., Baliki M.N. (2011). Pain and the brain: Specificity and plasticity of the brain in clinical chronic pain. Pain.

[5] Kucyi A., Davis K.D. (2015). The dynamic pain connectome. Trends Neurosci..

[6] Chen J., Wang X., Xu Z. (2023). The Relationship Between Chronic Pain and Cognitive Impairment in the Elderly: A Review of Current Evidence. J. Pain Res..

[7] Whitlock E.L., Diaz-Ramirez L.G., Glymour M.M., Boscardin W.J., Covinsky K.E., Smith A.K. (2017). Association Between Persistent Pain and Memory Decline and Dementia in a Longitudinal Cohort of Elders. JAMA Intern. Med..

[8] Wang J., Cheng Z., Kim Y., Yu F., Heffner K.L., Quiñones-Cordero M.M., Li Y. (2022). Pain and the Alzheimer’s Disease and Related Dementia Spectrum in Community-Dwelling Older Americans: A Nationally Representative Study. J. Pain Symptom Manag..

[9] Wang Z., Sun Z., Zheng H. (2024). Association between chronic pain and dementia: A systematic review and meta-analysis. Eur. J. Ageing.

[10] Huang F.F., Zhang Y., Liu L., Hsu C.L., Chung R., Wu W., Zheng D.K.Y., Xiong Z., Chang J.R., Zheng Y. (2025). The Longitudinal Association Between Chronic Back Pain and Cognitive Decline in Older Adults With Mediation Analysis: An Analysis of Four Population-Based Databases. Eur. J. Pain.

[11] Berryman A., Machado L. (2025). Cognitive Functioning in Females with Endometriosis-Associated Chronic Pelvic Pain: A Literature Review. Arch. Clin. Neuropsychol. Off. J. Natl. Acad. Neuropsychol..

[12] Bell T.R., Elman J.A., Gustavson D.E., Lyons M.J., Fennema-Notestine C., Williams M.E., Panizzon M.S., Pearce R.C., Reynolds C.A., Sanderson-Cimino M. (2025). History of chronic pain and opioid use is associated with cognitive decline and mild cognitive impairment. J. Int. Neuropsychol. Soc. JINS.

[13] Elliott J.H., Synnot A., Turner T., Simmonds M., Akl E.A., McDonald S., Salanti G., Meerpohl J., MacLehose H., Hilton J. (2017). Living systematic review: 1. Introduction—The why, what, when, and how. J. Clin. Epidemiol..

[14] Turner R.M., Davey J., Clarke M.J., Thompson S.G., Higgins J.P. (2012). Predicting the extent of heterogeneity in meta-analysis, using empirical data from the Cochrane Database of Systematic Reviews. Int. J. Epidemiol..

[15] Marshall I.J., Wallace B.C. (2019). Toward systematic review automation: A practical guide to using machine learning tools in research synthesis. Syst. Rev..

[16] Shamseer L., Moher D., Clarke M., Ghersi D., Liberati A., Petticrew M., Shekelle P., Stewart L.A. (2015). Preferred reporting items for systematic review and meta-analysis protocols (PRISMA-P) 2015: Elaboration and explanation. BMJ Br. Med. J..

[17] Page M.J., McKenzie J.E., Bossuyt P.M., Boutron I., Hoffmann T.C., Mulrow C.D., Shamseer L., Tetzlaff J.M., Akl E.A., Brennan S.E. (2021). The PRISMA 2020 statement: An updated guideline for reporting systematic reviews. BMJ.

[18] Treede R.-D., Rief W., Barke A., Aziz Q., Bennett M.I., Benoliel R., Cohen M., Evers S., Finnerup N.B., First M.B. (2019). Chronic pain as a symptom or a disease: The IASP Classification of Chronic Pain for the International Classification of Diseases (ICD-11). Pain.

[19] Cermelli A., Roveta F., Giorgis L., Boschi S., Grassini A., Ferrandes F., Lombardo C., Marcinno A., Rubino E., Rainero I. (2024). Is headache a risk factor for dementia? A systematic review and meta-analysis. Neurol. Sci..

[20] Yuan H., Ahmed W.L., Liu M., Tu S., Zhou F., Wang S. (2023). Contribution of pain to subsequent cognitive decline or dementia: A systematic review and meta-analysis of cohort studies. Int. J. Nurs. Stud..

[21] Zhu W., Zhan Y., Pei J., Fu Q., Wang R., Yang Q., Guan Q., Zhu L. (2025). Migraine is a risk factor for dementia: A systematic review and meta-analysis of cohort studies. J. Headache Pain.

[22] Bramer W.M., Giustini D., De Jonge G.B., Holland L., Bekhuis T. (2016). De-duplication of database search results for systematic reviews in EndNote. J. Med. Libr. Assoc. JMLA.

[23] Van De Schoot R., De Bruin J., Schram R., Zahedi P., De Boer J., Weijdema F., Kramer B., Huijts M., Hoogerwerf M., Ferdinands G. (2021). An open source machine learning framework for efficient and transparent systematic reviews. Nat. Mach. Intell..

[24] Higgins J.P.T., Morgan R.L., Rooney A.A., Taylor K.W., Thayer K.A., Silva R.A., Lemeris C., Akl E.A., Bateson T.F., Berkman N.D. (2024). A tool to assess risk of bias in non-randomized follow-up studies of exposure effects (ROBINS-E). Environ. Int..

[25] Schünemann H., Brożek J., Guyatt G., Oxman A. (2013). GRADE Handbook for Grading Quality of Evidence and Strength of Recommendations.

[26] Gelman A., Carlin J.B., Stern H.S., Rubin D.B. (1995). Bayesian Data Analysis.

[27] Gelman A. (2006). Prior distributions for variance parameters in hierarchical models (comment on article by Browne and Draper). Bayesian Anal..

[28] Bürkner P.-C. (2017). brms: An R package for Bayesian multilevel models using Stan. J. Stat. Softw..

[29] Lin L., Chu H. (2018). Quantifying publication bias in meta-analysis. Biometrics.

[30] Crippa A., Orsini N. (2016). Dose-response meta-analysis of differences in means. BMC Med. Res. Methodol..

[31] Greenland S., Longnecker M.P. (1992). Methods for trend estimation from summarized dose-response data, with applications to meta-analysis. Am. J. Epidemiol..

[32] Vehtari A., Gelman A., Gabry J. (2017). Practical Bayesian model evaluation using leave-one-out cross-validation and WAIC. Stat. Comput..

[33] Bartoš F., Gronau Q.F., Timmers B., Otte W.M., Ly A., Wagenmakers E.J. (2021). Bayesian model-averaged meta-analysis in medicine. Stat. Med..

[34] Ji R.-R., Nackley A., Huh Y., Terrando N., Maixner W. (2018). Neuroinflammation and central sensitization in chronic and widespread pain. Anesthesiology.

[35] Iliff J.J., Wang M., Liao Y., Plogg B.A., Peng W., Gundersen G.A., Benveniste H., Vates G.E., Deane R., Goldman S.A. (2012). A paravascular pathway facilitates CSF flow through the brain parenchyma and the clearance of interstitial solutes, including amyloid β. Sci. Transl. Med..

[36] Peng W., Achariyar T.M., Li B., Liao Y., Mestre H., Hitomi E., Regan S., Kasper T., Peng S., Ding F. (2016). Suppression of glymphatic fluid transport in a mouse model of Alzheimer’s disease. Neurobiol. Dis..

[37] Bushnell M.C., Čeko M., Low L.A. (2013). Cognitive and emotional control of pain and its disruption in chronic pain. Nat. Rev. Neurosci..

[38] Moriarty O., McGuire B.E., Finn D.P. (2011). The effect of pain on cognitive function: A review of clinical and preclinical research. Prog. Neurobiol..

[39] Meeus M., Goubert D., De Backer F., Struyf F., Hermans L., Coppieters I., De Wandele I., Da Silva H., Calders P. (2013). Heart rate variability in patients with fibromyalgia and patients with chronic fatigue syndrome: A systematic review. Semin. Arthritis Rheum..

[40] Gorelick P.B., Scuteri A., Black S.E., DeCarli C., Greenberg S.M., Iadecola C., Launer L.J., Laurent S., Lopez O.L., Nyenhuis D. (2011). Vascular contributions to cognitive impairment and dementia: A statement for healthcare professionals from the American Heart Association/American Stroke Association. Stroke.

[41] Borsook D. (2012). Neurological diseases and pain. Brain.

